# Color-advertising strategies of invasive plants through the bee eye

**DOI:** 10.3389/fpls.2024.1393204

**Published:** 2024-05-22

**Authors:** Martin Dessart, João Marcelo Robazzi Bignelli Valente Aguiar, Eric Tabacchi, Sylvie Guillerme, Martin Giurfa

**Affiliations:** ^1^ Institut de Recherche sur la Biologie de l'Insecte, Centre National de la Recherche Scientifique (CNRS), University of Tours, Tours, France; ^2^ Programa de Pós-Graduação em Entomologia, Faculdade de Filosofia, Ciências e Letras de Ribeirão Preto, Universidade de São Paulo, Ribeirão Preto, São Paulo, Brazil; ^3^ Centre de Recherche sur la Biodiversité et l'Environnement (CRBE), UMR 53000, Centre National de la Recherche Scientifique (CNRS), Institut de Recherche pour le Développement (IRD), Institut National Polytechnique, Université Paul Sabatier, Toulouse, France; ^4^ Laboratoire Géographie de l’Environnement (GEODE), Centre National de la Recherche Scientifique (CNRS), University Toulouse Jean-Jaurès, Toulouse, France; ^5^ Institut Universitaire de France, Paris, France; ^6^ Neuroscience Paris-Seine - Institut de Biologie Paris-Seine, Centre National de la Recherche Scientifique (CNRS), Institut National de la Santé et de la Recherche Médicale (INSERM), Sorbonne University, Paris, France

**Keywords:** invasive plants, honey bee, vision, color detection, color discrimination

## Abstract

Invasive plants represent a significant global challenge as they compete with native plants for limited resources such as space, nutrients and pollinators. Here, we focused on four invasive species that are widely spread in the French Pyrenees, *Buddleja davidii*, *Reynoutria japonica*, *Spiraea japonica* and *Impatiens glandulifera*, and analyzed their visual advertisement signals with respect to those displayed by their surrounding native species using a perceptual approach based on the neural mechanisms of bee vision given that bees are regular pollinators of these plants. We collected 543 spectral reflections from the 4 invasive species, and 66 native species and estimated achromatic and chromatic similarities to the bee eye. *R. japonica, S. japonica* and *B. davidii* were inconspicuous against the foliage background and could be hardly discriminated in terms of color from their surrounding native plants. These characteristics promote generalization, potentially attracting pollinators foraging on similar native species. Two morphs of *I. glandulifera* were both highly salient in chromatic and achromatic terms and different from their surrounding native species. This distinctive identity facilitates detection and learning in association with rich nectar. While visual signals are not the only sensory cue accounting for invasive-plant success, our study reveals new elements for understanding biological invasion processes from the perspective of pollinator perceptual processes.

## 1 Introduction

Invasive plant species represent a significant challenge in globalization times as they compete with native plants and animals for limited resources, modifying habitats and reducing biodiversity (IUCN Council, 2000; Charles and Dukes, 2007; Hejda et al., 2009; Pyšek et al., 2012; Barney et al., 2013; Langmaier and Lapin, 2020). Their success in certain biotopes indicates that some species rely on highly competitive traits allowing them to conquer and thrive in newly colonized ecosystems (Parker et al., 1999; Gioria and Osborne, 2014). Invasive plant species compete with native species for space, nutrients (Skurski et al., 2017) and pollinators (Traveset and Richardson, 2006; Bjerknes et al., 2007; Kovács-Hostyánszki et al., 2022). Several studies have revealed the disruption of native plant-pollinator networks by successful invasive plant species in detriment of native plant species, highlighting thereby one of the key factors of their success (Ghazoul, 2004; Bjerknes et al., 2007; Bartomeus et al., 2008; Goodell and Parker, 2017; Stout and Tiedeken, 2017; Burns et al., 2019; Parra-Tabla and Arceo-Gomez, 2021; Kovács-Hostyánszki et al., 2022). Some invasive plants may provide richer nectar, being therefore particularly attractive for pollinators (Chittka and Schurkens, 2001), or more conspicuous flowers that outcompete those of native plants (Traveset and Richardson, 2006; Sooraj et al., 2019). They are also usually generalists with respect to pollinators, a strategy that allows them benefiting from the fertilization contribution of local pollinators (Richardson et al., 2007; Parra-Tabla and Arceo-Gomez, 2021). Abundant invasive plants can even dominate the plant-pollinator network, to a point where the interaction between native plants and their pollinators is modified (Albrecht et al., 2014).

Color is one of the main advertising cues displayed by flowers to pollinators (Kevan, 1983; Weiss, 1991; Menzel and Shmida, 1993; Chittka et al., 1999). Among the main pollinators of flowers, the western honey bee (*Apis mellifera* L.) is the most frequent floral visitor of crops worldwide (Hung et al., 2018; Khalifa et al., 2021). Honey bees are also one of the insects best studied in terms of their visual perception, from behavior to the neural underpinnings of visual performances (Giurfa and Lehrer, 2001; Avarguès-Weber et al., 2011; Avarguès-Weber et al., 2012; Mota et al., 2013; Avarguès-Weber and Giurfa, 2014). Bees possess trichromatic color vision based on three photoreceptors types with sensitivities peaking at 344 nm in the short-wave (ultra violet) region of the spectrum, 436 nm in the middle-wave (blue) region, and 544 nm in the long-wave (green) region of the spectrum (L receptor), respectively (Menzel and Blakers, 1976; Peitsch et al., 1992). Photoreceptor signals are fed into color-opponent neurons present in higher-order visual areas of the bee brain (Kien and Menzel, 1977; Backhaus, 1991), giving origin to color sensations. Color processing networks are therefore well-characterized in the honey bee, which provided the basis for the conception of color perceptual spaces that allow determining to what extent colors are discriminable for a honey bee (Backhaus, 1991; Chittka, 1992). This strategy has been used to assess flower color perception and discrimination by bees in multiple studies e.g. (Kevan et al., 1996; Reisenman and Giurfa, 2008; Arnold et al., 2009; Aguiar et al., 2020; Shrestha et al., 2024).

However, few analyses have focused on the floral color of invasive species and its role and contribution to their ecological success from a pollinator vision perspective. Analyses performed on an Indian community including 22 native and 8 invasive plant species showed that invasive species with higher UV absorbance tended to differ chromatically from native species, a strategy that may increase their attractiveness and discriminability for pollinators (Sooraj et al., 2019). Yet, the strategy adopted by invasive plants may vary significantly according to the characteristics of their ecological niche and those of the surrounding native species.

The region of the Pyrenees, delimiting the border between France and Spain, has been subjected to intensive invasion by various plant species (Guillerme et al., 2020; Claudel et al., 2022). The area is also rich and reputed for the diversity of local flora (Saule, 2002) and the presence of numerous bee pollinating species so that it provides an attractive and relevant scenario to study the color strategies used by invasive plant species. Understanding the role of floral coloration through the bee eye is a powerful proxy for understanding the success of invasive plants’ reproduction. We thus focused on four dominant invasive plant species that can be found in the French Pyrenees, the butterfly bush (*Buddleja davidii*, Scrophulariaceae), the Japanese Knotweed (*Reynoutria japonica*, Polygonaceae), the Japanese Spirea (*Spiraea japonica*, Rosaceae) and the Himalaya Balsam (*Impatiens glandulifera*, Balsaminaceae), which are frequently visited by honey bees (Ellis and Ellis-Adam, 1993; Najberek et al., 2023). We studied how their color displays are perceived by bee pollinators in comparison to those of surrounding native species. To this end, we used perceptual neural modelling of honey bee vision, which allowed us to analyze the color advertising strategies of invasive species as seen by the bee eye. While an integrative analysis of invasive-plant success should include measures of plant visitation by pollinators and of plant fitness, our goal here was to rely on a neuroscience approach to provide evidence on pollinator perception that could serve as guide for future works by scholars working in ecology, plant science and other related disciplines.

Our results show that the color advertising strategies of the four species considered are heterogeneous as some of them display colors that are similar to those of surrounding native species, potentially promoting generalization, while others display highly prominent color and achromatic cues different from those of the surrounding native species to facilitate color detection and learning by pollinators.

## 2 Materials and methods

### 2.1 Study area and invasive plant species

**Figure 1:**
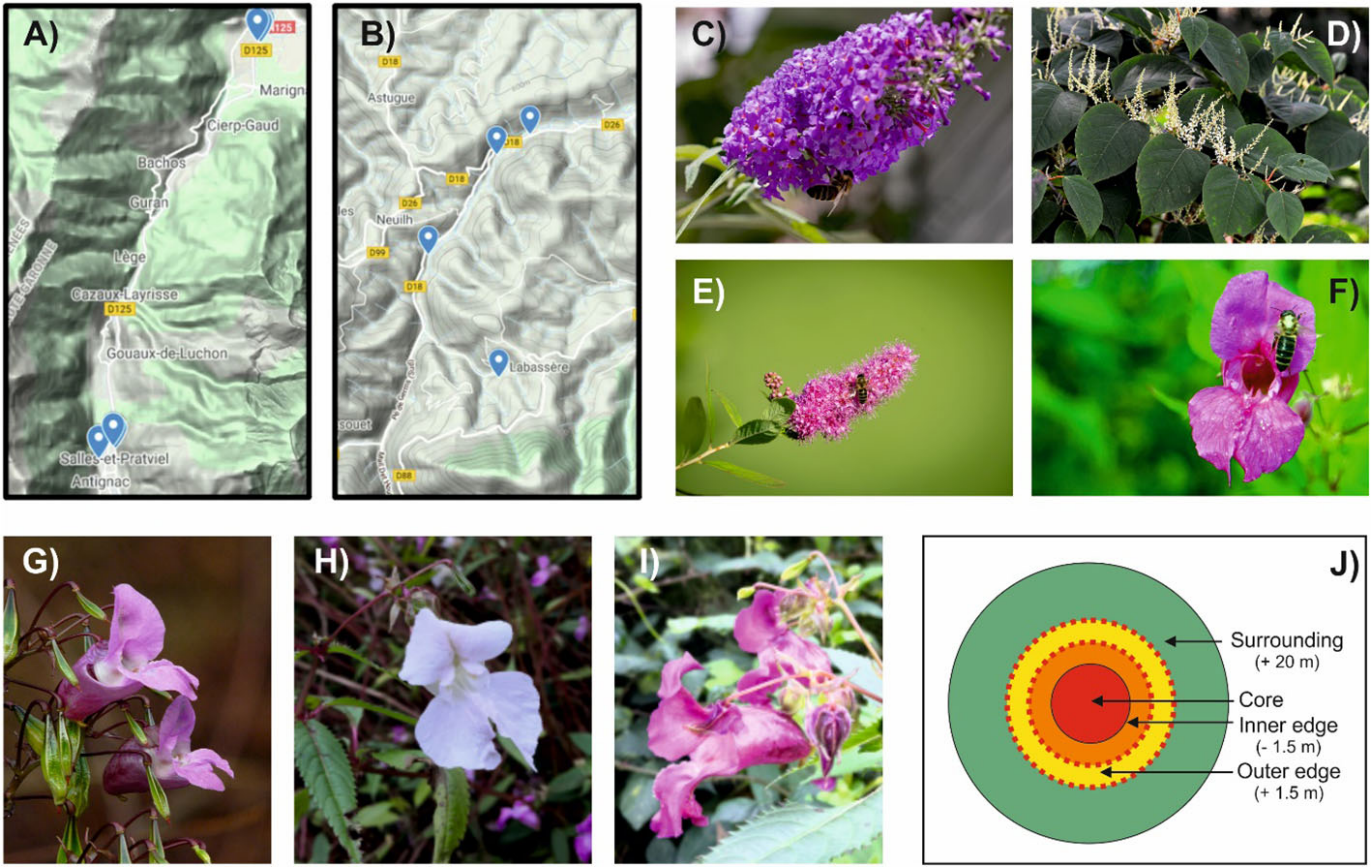
Study areas, invasive species studied and sampling strategy. **(A)** Relief map of the Pique Valley (42°45’ N, 0°37’ E, 1200 m mean altitude), one of the areas chosen for our study. **(B)** Relief map of the Oussouet Valley (43°5’ N, 0°5’ E, 800 m mean altitude), the other area chosen for our study. **(C)** The invasive butterfly bush (*Buddleja davidii* Franch). **(D)** The invasive Japanese Knotweed (*Reynoutria japonica* Houtt). **(E)** The invasive Japanese Spirea (*Spiraea japonica* L.). **(F)** The invasive Himalaya Balsam (*Impatiens glandulifera* Royle). The latter included three different morphs, which appeared violet **(G)**, white **(H)** and pink **(I)** to the human eye. **(J)** Sampling strategy of permanent vegetation patches centred on a particular hotspot of a specific invasive plants. Within each patch, four concentric zones were established. The ‘*core zone*’ was densely invaded by one of the invasive species chosen for our study. The ‘*inner edge zone*’ was delineated as an inner ring within the core zone (~1,5 m wide strip) using the average quasi-absence (< 5% cover) of the invader as criterion. The ‘*outer edge zone*’ was defined as an external ring surrounding the core zone (~1,5 m wide strip) in which the invader was totally absent. Finally, the more eccentric ‘*surrounding zone*’ was defined as a broader area included within a 20m width strip beyond the outer edge zone. For each plant species, we estimated visually the relative cover within each zone.

We investigated two sites located at the foothills of the Central Pyrenees Mountains (SW France, **Figure 1**): the Pique Valley (42°45’ N, 0°37’ E, 1200 m mean altitude) (**Figure 1A**) and the Oussouet Valley (43°5’ N, 0°5’ E, 800 m mean altitude) (**Figure 1B**). Both have very similar landscapes including agro-pastoral and forested units hosting high levels of plant and animal biodiversity (Rhoné, 2015), including abundant invasive species (Tabacchi et al., 2010; Guillerme et al., 2020; Jantzi et al., 2021). The climate of the sampled sites corresponds to mountain-oceanic temperate conditions (Mean temperature: 4,0 – 16,5°C; Monthly accumulated precipitation: 27,8mm – 174,2mm).

The dominant invasive species found in the study areas were the butterfly bush (*Buddleja davidii* Franch.; **Figure 1C**), the Japanese Knotweed (*Reynoutria japonica* Houtt.; **Figure 1D**), the Japanese Spirea (*Spiraea japonica* L.*;* **Figure 1E**) and the Himalaya Balsam (*Impatiens glandulifera* Royle; **Figure 1F**), which provided the basis for our study. The latter included three different morphs, which appeared violet (**Figure 1G**), white (**Figure 1H**) and pink (**Figure 1I**) to the human eye.

We conducted our surveys at four distinct dates - 20^th^ and 22^nd^ of July 2020, 23^rd^ and 28^th^ of September 2020 - which corresponded to the average maximum development of the vegetation.

For each sampling session, we chose 12 representative permanent vegetation patches centered on a particular hotspot of a specific invasive plants among the four species targeted. Each patch corresponded to one invasive species; in total, we established 2 patches for *B. davidii*, 3 patches for *R. japonica*, 1 patch for *S. japonica* and 2 patches for each morph of *I. glandulifera* (6 patches for *I glandulifera*). Each patch corresponded to a particular site in order to integrate spatial variability. Data were pooled as an analysis per patch would require a higher number of replicates, which was not easily accessible. At the regional scale, these sampling units were selected based on an aerial photograph survey. The location of each patch was confirmed by local preliminary field surveys. Within each identified patch, we delineated four concentric zones (**Figure 1J**). The ‘*core zone*’ was densely and almost exclusively occupied by one of the invasive species chosen for our study. The ‘*inner edge zone*’ was delineated as an inner ring within the core zone (~1,5 m wide strip) using the average quasi-absence (< 5% cover) of the invader as criterion. The ‘*outer edge zone*’ was defined as an external ring surrounding the core zone (~1,5 m wide strip) in which the invader was totally absent. Finally, the more eccentric ‘*surrounding zone*’ was defined as a broader area included within a 20m width strip beyond the outer edge zone. By definition the more external zone corresponds to areas where the invader was absent. This is a consequence of the patchy pattern induced by the competition/colonization processes, but probably also by the effect of management (mowing) in the outer zone.

### 2.2 Reflectance measurements and analysis

A minimum of three fresh flowers of each species were systematically collected at each patch along the four zones in order to measure their spectral reflectance. Reflectance measurements in the bee visible range (300 – 650 nm) (Menzel and Blakers, 1976) were performed *in situ* and with a resolution of 1 nm using a UV-VIS Ocean Optics USB400 spectrometer (Dunedin, Florida, USA) and a pulsed Xenon Lamp PX-2 light source. A white standard (barium sulphate) was used to calibrate the spectrophotometer before each session of measurements. The reflectance spectra of 70 plant species were included in our analyses (see **Supplementary Table S2**). Reflectance spectra were obtained from 50 native species (n = 333 spectra; 1 spectrum corresponding to 1 flower part) and from the 4 invasive species mentioned above (n = 102 spectra), all collected in the field, in the areas defined for our sampling. The FRED database (Arnold et al., 2010) allowed us to include 16 additional native species (n = 34 spectra) to complement our measurements (**Supplementary Table S2**). These species were present in the field and were counted for abundancy measurements but were inaccessible for reflectance measurements. The same database was used to include complementary measurements for 32 native species (n = 74 spectra) that were also sampled in the field (see **Supplementary Table S2** for details). Overall, our analyses included 543 spectra (435 from the field and 108 from the FRED data), from 70 plant species, including the 4 invasive species, which were the focus of our study, and 66 native species (18 exclusively from the field, 16 exclusively from the FRED data base, and 32 both from the field and the FRED data base). For all species, both spectral curves and relative abundance measurements were available and used for our analyses. We also measured the spectral reflectance from leaves of 15 species (n = 25 spectra) to characterize the green foliage background (see **Supplementary Table S2**).

### 2.3 Color perception models

We used the *pavo* package (Maia et al., 2013) to normalize reflectance spectra in the wavelength range comprised between 300-650 nm. Curves were smoothed to remove noise. Flower reflectance curves were averaged within each species. All statistical analyses were conducted using R software version 4.0.4 (https://www.r-project.org/).

To test differences in flower colour according to pollinator vision, floral reflectance data were transformed into color loci within the color hexagon, a perceptual space allowing the evaluation of flower colors as seen by honey bees, which are dominant pollinators in the regions of interest (Chittka, 1992). For calculations, we used the standard illuminant function D65, the average function obtained from green leaves sampled as the background and the spectral sensitivity curves of the three photoreceptor types of the honey bee *Apis mellifera* (Peitsch et al., 1992). We created the coldspace object by passing the vismodel object and selected the hexagon to plot our models. To calculate color distances, we passed the coldspace object and obtained unweighted Euclidean distances. All the data and code used are available in: https://github.com/martindessart/Invasive_plants_through_bee-eye_Pyrenees.

### 2.4 Data clustering and PCA analysis

In order to evaluate the color similarity of invasive and native flower species to the honey bee eye, we first calculated indices to determine the optimized number of clusters of flower loci within the hexagon space. These clusters were used to describe our dataset into predefined groups that have similar properties.

We first used the NbClust package from R software (Charrad et al., 2014), which provides 30 indices and allows choosing the best clustering size for our data set. We verified this choice using the function fviz_nbclust associated with the silhouette method from the FactoExtra package (Kassambara and Mundt, 2020). We ran a PCA analysis based on the coordinates of the flower loci in the hexagon space using k-means clustering. This method of vector quantization partitioned our data into k groups that minimized the sum of squares from points to the assigned cluster center (see **Supplementary Material**). All statistical analyses were conducted using R software version 4.0.4 (https://www.r-project.org/).

### 2.5 Question 1: do invasive floral species have more salient visual cues to the bee eye?

For each flower species, we calculated the chromatic contrast, the achromatic contrast and the spectral purity. The chromatic contrast refers to the perceptual difference between the color of the flower and that of the background. Given that the color hexagon is a bidimensional space that includes only the dimensions of hue and saturation (i.e. no brightness dimension), the chromatic contrast can be quantified as the distance between the locus of a flower species in that space and that of the background. The latter occupies the center of the space (0, 0 coordinates) as it acts as the adaptation background against which colors are evaluated (Chittka, 1992). Chromatic contrast involves true color vision and allows close-up detection of color targets with larger visual angles, i.e. typically larger than 15° (Giurfa et al., 1996b; Giurfa et al., 1997). Chromatic differences between color loci are quantified in terms of the Euclidian distance between two color loci (see below); in the hexagon this distance is given in hexagon units HU (Chittka, 1992).

The achromatic contrast refers to the relative number of absorbed quanta by the L-receptor type (longwave or ‘green’ photoreceptor type) upon stimulation with the flower color with respect to the background. This receptor-specific contrast provides an achromatic channel allowing long-distance detection of visual targets (i.e. targets with a reduced visual angle, typically smaller than 15°) (Giurfa et al., 1997; Giurfa and Vorobyev, 1998).

Finally, the spectral purity represents the saturation of a given color, a variable that may affect floral preference in bees (Lunau, 1990; Lunau, 1992; Lunau et al., 2006; Rohde et al., 2013). It is quantified by dividing the distance between the loci of the floral color and the background (chromatic contrast, see above) by the distance between the corresponding monochromatic light with the same dominant wavelength as the color target and the background (Lunau et al., 1996).

Two steps were designed for the analysis of flower salience. First, group-level salience was assessed by comparing invasive and native flowers in terms of three variables defined above (i.e., chromatic contrast, achromatic contrast and spectral purity) using a one-sample Kruskal-Wallis H test with group as the fixed factor. To analyze the salience of one invasive species with respect to groups of native species, a one-sample Wilcoxon signed-rank test was used setting the invasive-species value of the variable considered as reference for the comparison. This analysis was also performed segregating groups and invasive species according to the sample areas (core, inner and outer edges and surrounding).

### 2.6 Question 2: are invasive flowers species discriminable from native species to the bee eye?

We followed the procedure from Maia and White (2018) to determine if colors were discriminable from each other. The method considers the statistical distribution, the within-group variation and the discriminability of groups based on honey bee visual abilities. A distance-based PERMANOVA using the Euclidian distances between species in the hexagon was applied to two groups of interest (e.g., groups determined by the clustering analysis) to determine if they were significantly distinct. A bootstrap procedure was then applied to simulate new samples, in order to obtain a distribution of the mean distance between each sample and the group geometric mean, and thus a 95% confidence interval for each color comparison. We finally compared the values obtained for each invasive species to the bees’ color discrimination threshold of 0.1 hexagon units (HU), which has been reported for bees trained under absolute conditioning (a single color rewarded) (Dyer and Chittka, 2004). This kind of training corresponds to the ecological scenario of a flower-constant pollinator visiting a single species during its foraging bouts. If a color distance was lower than this threshold, we concluded that the samples compared were perceptually similar and thus indistinguishable in chromatic terms by honey bees (Maia and White, 2018).

## 3 Results

### 3.1 Spectral reflectance curves and flower colors in perceptual color spaces

**Figure 2:**
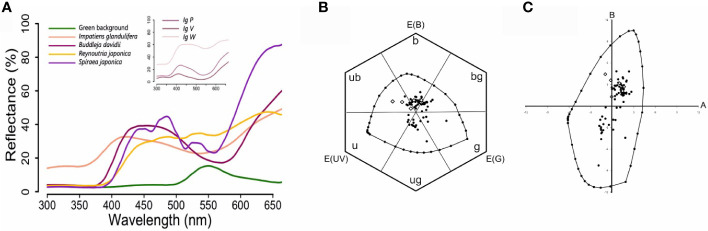
**(A)** Spectral reflectance curves of the four invasive floral species studied. Normalized average reflectance (%) of *Buddleja davidii*, *Reynoutria japonica, Spiraea japonica* and *Impatiens glandulifera.* Royle in the visible range of the honey bee *Apis mellifera* (from 300 to 650 nm). The average reflectance of green leaves used as background for further perceptual analyses of floral colors is also shown. The inset shows the normalized average reflectance (%) of the three morphs of *I. glandulifera*, *IgP* (pink), *IgV* (violet) and *IgW* (white). **(B)** Color loci of the flower species analyzed in the color hexagon. The colour hexagon is a generalized color opponent space with metrics applicable to numerous species of Hymenoptera. The average reflectance of green leaves was used as background and corresponds to the center of the hexagon. Photoreceptor excitations E(UV), E(B), E(G) are plotted at angles of 120°. The hexagon is divided in six segments corresponding to six categories, which refer to the ways in which the bees’ receptors are stimulated by given pure broad-band spectral stimuli, or with uv-green, mixed spectral stimuli: u (ultraviolet), ub (ultraviolet-blue), b (blue), bg (blue-green), g (green) and ug (ultraviolet-green). The loci within the space correspond to the 66 native species (black dots) and the 4 invasive species, one of which had three different morphs (white diamonds), i.e. 72 color loci represented. The closed line surrounding the floral color loci defines the boundaries of color perception at adaptation light. The loci along the spectral line are marked in 10 nm steps, and the mixtures of 300 and 550 nm (ultraviolet-green) in 10% steps. **(C)** Color loci of the flower species included in our analyses in the color opponent coding (COC) space. The COC space is a color opponent space in which floral color loci of invasive species, native species of Group1 and native species of Group 2 are plotted as a function of the responses of two types of colour opponent coding cells, A and B. The origin of the graph represents the green leaves used as background. The loci within the space correspond to the 66 native species (black dots) and the 4 invasive species, one of which had three different morphs (white diamonds), i.e. 72 color loci represented. The closed line surrounding the floral color loci defines the boundaries of color perception at adaptation light. The loci along the spectral line are marked in 10 nm steps, and the mixtures of 300 and 550 nm (ultraviolet-green) in 10% steps.

We measured the spectral reflectance of the four invasive species considered in our study, *Reynoutria japonica*, which was whitish to the human eye, *Buddleja davidii*, which was violet-pinkish to the human eye, *Spiraea japonica* L., which was pink to the human eye (**Figure 2A**), and *Impatiens glandulifera*, which presented three different colored morphs that appeared violet, white and pink to the human eye (inset of **Figure 2A**). In addition, we measured the average reflectance of green leaves present in the sampling areas (**Figure 2A**) in order to use it as the background for representing flower colors in perceptual color spaces. Our measurements allowed us to represent the loci of the invasive species, together with those of all flower species included in our study, in the perceptual spaces of the color hexagon (**Figure 2B**) and the color opponent coding space (**Figure 2C**) defined for the honey bee. Subsequent analyses on flower color loci were restricted to the color hexagon given the higher generality of this perceptual space for pollinators other than honey bees (Backhaus, 1991; Chittka, 1992).

### 3.2 Data clustering and PCA analysis

**Figure 3:**
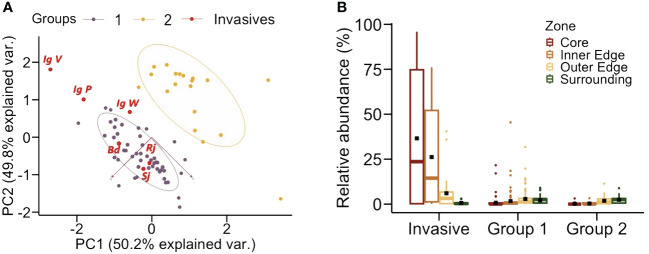
**(A)** Clustering analysis of the floral data set used in this study. Unsupervised hierarchical clustering performed on our flower data set allowed to distinguish two groups according to their color loci in the hexagon. The two axes indicate in parentheses the % of the data variance accounted for by our analysis. Group 1 (violet dots) included 52 species: 48 native species and the 4 invasive species Bd (*Buddleja davidii*), Sj (*Spiraea japonica*), Rj (*Reynoutria japonica*) and the three morphs of Ig (*Impatiens glandulifera*) IgV, IgW and IgP, all located in the bee ultraviolet-blue and blue regions of the hexagon. Invasive species are shown by red dots. Group 2 (yellow dots) included 18 native species located in the ultraviolet-green and green regions of the hexagon. **(B)** Relative frequency of the invasive species and the two groups of native species in each sampled zone. Per definition, invasive species were mostly located in the core zone, as sampling areas were centered on a particular hotspot of an invasive plant species. They could also be located in the inner edge zone among the four species targeted. Group 1 species were mostly found in the inner edge and in the outer edge zone. Group 2 species were less frequent and usually located in the outer edge and the surrounding zones. Boxplots show the median (horizontal line) and interquartile ranges. Bars indicate +/- interquartile ranges. Dark squares indicate mean value for the zone considered.

Unsupervised hierarchical clustering was performed on our flower data set to group species by color. First, we assessed the clustering tendency by calculating the Hopkins statistic using the *get_clust_tendency* function from the FactoExtra R package (Kassambara and Mundt, 2020). The closer the Hopkins statistic is to 1, the more the data are clustered (Jove et al., 2020). In our case, the value of 0.726 indicated that the dataset contained suitable information for clustering. We then performed two independent analyses to validate the correct number of clusters using the NbClust package and the FactoExtra package. The data clustering analysis separated two distinct groups according to their color loci (**Figure 3A**).

The first group (hereafter Group 1) included 52 species (48 native species and the 4 invasive species), which were located in the bee UV-blue and blue regions of the hexagon (human blue – purple) (**Figure 2B**). The second group (hereafter Group 2) included 18 native species, which were located in the bee ultraviolet-green and green regions of the hexagon (**Figure 2B**). Invasive flower species were located per definition in the core zone as sampling patches were centered on a particular hotspot of an invasive plant species (**Figure 3B**). They could also bee found in the inner edge zone. Group-1 native species were mostly found close to patch core zones, i.e. in the inner and the outer edge zones. Group-2 native species were less frequent and usually located in the outer edge and the surrounding zones of the designed plots in the field (**Figure 3B**).

### 3.3 Question 1: do invasive floral species have more salient visual cues to the bee eye?

**Figure 4:**
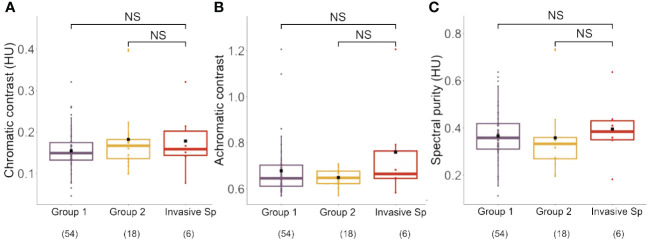
**(A)** Chromatic contrast (hexagon Units, HU) of Group1, Group 2 and the invasive species analyzed in our study. No significant differences between groups were found for this parameter. **(B)** Achromatic contrast (L-receptor contrast with respect to the background) of Group1, Group 2 and the invasive species analyzed in our study. No significant differences between groups were found for this parameter. **(C)** Spectral purity (hexagon Units, HU) of Group1, Group 2 and the invasive species analyzed in our study. No significant differences between groups were found for this parameter. Boxplots show the median values (thick line) and interquartile ranges; dots indicate values recorded for each species; black squares indicate mean values of each group. Number in parentheses indicate sample sizes. NS, not significant.

We calculated the average chromatic contrast, achromatic contrast and spectral purity for the four invasive species and for the native species of Group 1 and Group 2 (**Figures 4A–C**; see **Supplementary Table S3** for complete set of values).

Chromatic contrast calculations based on the visual system of honey bees yielded values ranging from 0.05 to 0.40 hexagon units (HU) (**Figure 4A**; Group 1: 0.15 ± 0.05; Group 2: 0.18 ± 0.08; Invasive Species: 0.18 ± 0.08; mean ± SD), which did not differ significantly between groups (Kruskal Wallis Test: χ^2 ^= 1.69, df: 2 p = 0.43). The calculation of achromatic contrast yielded values ranging from 0.57 to 1.21 (**Figure 4B**; Group 1: 0.68 ± 0.12; Group 2: 0.65 ± 0.03; Invasive Species: 0.76 ± 0.23; mean ± SD), which did also not differ between groups (χ^2 ^= 0.86, df:2, p = 0.65). Finally, spectral purity values, which ranged from 0.11 to 0.73 HU (**Figure 4C**; Group 1: 0.37 ± 0.11; Group 2: 0.36 ± 0.15; Invasive Species: 0.40 ± 0.15; mean ± SD; see **Supplementary Table S3**), did also not differ between groups (χ^2 ^= 1.78, df = 2; p = 0.41). These results indicate that when taken together the four invasive species did not differ in chromatic and achromatic salience to bee eye. Yet, as the four invasive species differed in their visual display, averaging their values for a between-category analysis may hide significant trends at a single-species level. We thus performed separate comparisons between each invasive species and the two groups of native species defined in our cluster analysis.

While no clear differentiation trend was observable for *B. davidii, R. japonica* and *S. japonica* in terms of chromatic contrast, spectral purity and achromatic contrast, highly significant differences were found between the *I. glandulifera* morphs and Group 1 and 2 of native species (**Table 1**). While the violet (Ig V) and the pink (Ig P) morphs presented significantly higher chromatic and achromatic contrasts, as well as a higher spectral purity than native species in Groups 1 and 2 (p < 0.0001 for 10 of the 12 comparisons, and p < 0.03 for the remaining two comparison; see **Table 1**), the white morph (Ig W) had significantly lower values for the three variables considered than native species in Groups 1 and 2 (p < 0.0001 for all 6 comparisons; see **Table 1**). This analysis thus shows that three of the invasive species, *B. davidii, R. japonica* and *S. japonica*, which clustered with native species of Group 1 (**Figure 3**), did not differ particularly from their surrounding native species while *I. glandulifera* morphs, which were outside that cluster (**Figure 3**), adopted a different displaying strategy to the bees’ eyes: the violet and the pink morphs appeared more salient and thus better detectable to pollinators than native species, while the white morph was less salient than the native species.

**Table 1:** Chromatic contrast, achromatic contrast and spectral purity of single invasive species compared to values of Group 1 and Group 2 of native species.

	Comparisons with Group 1									Comparisons with Group 2								
	Chromatic contrast			Achromatic contrast			Spectral purity			Chromatic contrast			Achromatic contrast			Spectral purity		
	V statistic	n	p	V statistic	n	p	V statistic	n	p	V statistic	n	p	V statistic	n	p	V statistic	n	p
**Ig V**	0	54	< 0.0001	0	54	< 0.0001	0	54	< 0.0001	3	18	< 0.0001	0	18	< 0.0001	3	18	< 0.0001
**Ig P**	83	54	< 0.0001	117	54	< 0.0001	244	54	< 0.0001	36	18	0.03	0	18	< 0.0001	35	18	0.03
**Ig W**	1423	54	< 0.0001	1420	54	< 0.0001	1423	54	< 0.0001	171	18	< 0.0001	170	18	< 0.0001	171	18	< 0.0001
**Bd**	698	54	0.88	479	54	0.04	727	54	0.92	113	18	0.25	20	18	< 0.01	51	18	0.14
**Rj**	930	54	0.06	867	54	0.18	829	54	0.32	130	18	0.05	100	18	0.55	66	18	0.42
**Sj**	446	54	0.02	836	54	0.29	365	54	< 0.01	83	18	0.93	98	18	0.61	36	18	0.03

To analyze the salience of a single invasive species with respect to Groups 1 and 2 of native species, a one-sample Wilcoxon signed-rank test was used setting the invasive-species value of the variable considered as reference for the comparison. V statistic values, sample sizes (n) of the Group and p values are provided for each comparison performed; df: 1 for all comparisons. Ig V, violet morph of *Impatiens glandulifera;* Ig P, pink morph of *Impatiens glandulifera; Ig W, violet morph of Impatiens glandulifera; Bd, Buddleja davidii; Rj, Reynoutria japonica; Sj, Spiraea japonica.* Values in red indicate significant P values.

**Figure 5:**
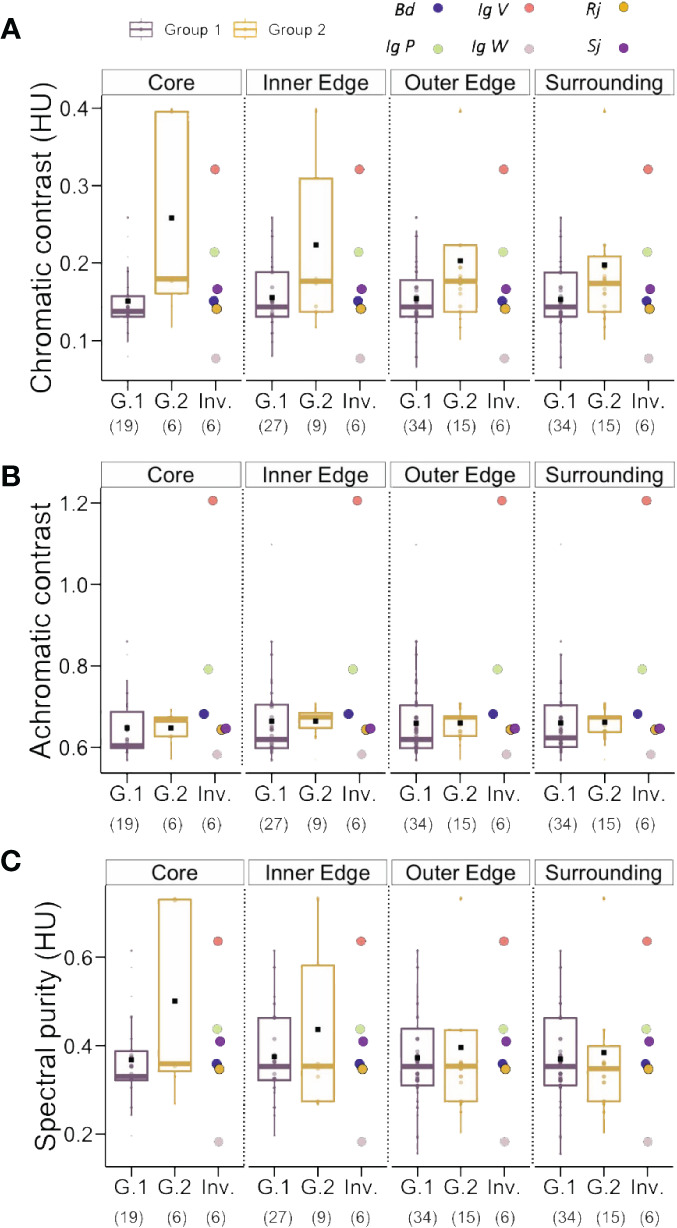
**(A)** Chromatic contrast (hexagon Units, HU), **(B)** Achromatic contrast (L-receptor contrast with respect to the background) and **(C)** Spectral purity (hexagon Units, HU) of Group1 (G.1), Group 2 (G.2) and the invasive species (Inv.) analyzed according to the sampling zones, core, inner and outer edges and surrounding zone. Bd, *Buddleja davidii*; Sj, *Spiraea japonica*; Rj, *Reynoutria japonica*; Ig, *Impatiens glandulifera*; IgV, IgW and IgP, violet, white and pink morphs of Ig. Number in parentheses indicate sample sizes.

In order to refine this analysis, we repeated the previous analysis of chromatic and achromatic contrasts and spectral purity but this time segregating the data according to the sampling zones defined in **Figure 1J**. **Figures 5A–C** and **Table 2** show the three variables evaluated (chromatic contrast, achromatic contrast and spectral purity, respectively) for Groups 1 and 2 of native species and for the invasive species as a function of the sampling zone. Some significant differences were found for the three variables when invasive species were compared to Group 2 of native species (see **Table 2**, right), in particular in the case of the achromatic contrast. Yet, the interesting comparisons are those between invasive species and Group 1 of native plants as relative-abundance analyses (see **Figure 3**) showed that Group 1 species tended to be more present around invasive species in the central sampling areas (core, inner and outer edge) than Group 2 species. **Figure 5** and **Table 2** show that while *R. japonica* did neither differ from its surrounding native species in chromatic contrast nor in achromatic contrast nor in spectral purity, the other invasive species showed either partial (for one or two variables) or total (for all three variables) significant differences with native species of Group 1. *B. davidii*, for instance, had a higher achromatic contrast than native species of Group, 1 which favors long-distance detection (Giurfa et al., 1997), while *S. japonica* was more salient in terms of chromatic variables such as chromatic contrast and spectral purity. *I. glandulifera* morphs presented consistent significant differences in all three variables when compared to surrounding native species of Group 1. The violet and the pink morphs (IgV and IgP) had significantly higher achromatic and chromatic contrasts and spectral purity than surrounding native species, which provides advantages in terms of farther visual detection and higher chromatic salience which may favour better associative learning by pollinators. Interestingly the opposite trend was found for the white morph (IgW), which had significantly lower values for all three variables.

**Table 2:** Chromatic contrast (A), achromatic contrast (B) and spectral purity (C) of single invasive species compared to values of Group 1 and Group 2 of native species according to the sample zone (Core, Inner Edge, Outer Edge and Surrounding).

A)	Chromatic Contrast																							
	Comparisons with Group 1												Comparisons with Group 2											
	*Core*			*Inner Edge*			*Outer Edge*			*Surrounding*			*Core*			*Inner Edge*			*Outer Edge*			*Surrounding*		
	V statistic	n	p	V statistic	n	p	V statistic	n	p	V statistic	n	p	V statistic	n	p	V statistic	n	p	V statistic	n	p	V statistic	n	p
***Ig V***	0	33	< 0.0001	0	54	< 0.0001	0	82	< 0.0001	0	86	< 0.0001	6	6	0,2	10	14	< 0.01	15	30	< 0.0001	21	38	< 0.0001
***Ig P***	16	33	< 0.0001	63	54	< 0.0001	135	82	< 0.0001	121	86	< 0.0001	18	6	0,55	57	14	0,89	155	30	0,07	229	38	0,02
***Ig W***	595	33	< 0.0001	1540	54	< 0.0001	3482	82	< 0.0001	3819	86	< 0.0001	28	6	0,02	120	14	< 0.001	496	30	< 0.0001	780	38	< 0.0001
***Bd***	232	33	0,27	746	54	0,84	1622	82	0,58	1825	86	0,71	23	6	0,15	93	14	0,06	395	30	< 0.01	582	38	< 0.01
***Rj***	332	33	0,56	971	54	0,09	2090	82	0,12	2340	86	0,07	26	6	0,05	100	14	0,02	431	30	< 0.001	647	38	< 0.001
***Sj***	152	33	0,01	496	54	0,02	1093	82	< 0.01	1147	86	< 0.01	21	6	0,27	81	14	0,24	317	30	0,18	444	38	0,45
B)	Achromatic Contrast																							
	Comparisons with Group 1												Comparisons with Group 2											
	*Core*			*Inner Edge*			*Outer Edge*			*Surrounding*			*Core*			*Inner Edge*			*Outer Edge*			*Surrounding*		
	V statistic	n	p	V statistic	n	p	V statistic	n	p	V statistic	n	p	V statistic	n	p	V statistic	n	p	V statistic	n	p	V statistic	n	p
***Ig V***	0	33	< 0.0001	0	54	< 0.0001	0	82	< 0.0001	0	86	< 0.0001	0	6	0,02	0	14	< 0.001	0	30	< 0.0001	0	38	< 0.0001
***Ig P***	15	33	< 0.0001	88	54	< 0.0001	138	82	< 0.0001	135	86	< 0.0001	0	6	0,02	0	14	< 0.001	0	30	< 0.0001	0	38	< 0.0001
***Ig W***	584	33	< 0.0001	1523	54	< 0.0001	3463	82	< 0.0001	3809	86	< 0.0001	27	6	0,03	119	14	**< 0.001**	495	30	< 0.0001	779	38	< 0.0001
***Bd***	142	33	< 0.01	466	54	0,01	933	82	< 0.001	1047	86	< 0.001	3	6	0,08	36	14	0,18	103	30	< 0.01	162	38	< 0.01
***Rj***	268	33	0,62	851	54	0,5	1845	82	0,65	2098	86	0,44	18	6	0,55	98	14	**0,03**	377	30	0,01	622	38	< 0.01
***Sj***	266	33	0,6	833	54	0,6	1793	82	0,82	2034	86	0,61	18	6	0,55	98	14	**0,03**	376	30	0,01	621	38	< 0.01
C)	Spectral Purity																							
	Comparisons with Group 1												Comparisons with Group 2											
	*Core*			*Inner Edge*			*Outer Edge*			*Surrounding*			*Core*			*Inner Edge*			*Outer Edge*			*Surrounding*		
	V statistic	n	p	V statistic	n	p	V statistic	n	p	V statistic	n	p	V statistic	n	p	V statistic	n	p	V statistic	n	p	V statistic	n	p
***Ig V***	0	33	< 0.0001	0	54	< 0.0001	0	82	< 0.0001	0	86	< 0.0001	6	6	0,2	10	14	< 0.01	15	30	< 0.0001	21	38	< 0.0001
***Ig P***	109	33	< 0.01	310	54	< 0.001	750	82	< 0.0001	711	86	< 0.0001	18	6	0,55	54	14	0,75	145	30	0,04	219	38	0,02
***Ig W***	595	33	< 0.0001	1540	54	< 0.0001	3482	82	< 0.0001	3819	86	< 0.0001	28	6	0,02	120	14	< 0.001	496	30	< 0.0001	780	38	< 0.0001
***Bd***	260	33	0,53	793	54	0,85	1740	82	0,99	1918	86	0,99	19	6	0,45	66	14	0,75	228	30	0,7	310	38	0,27
***Rj***	323	33	0,67	894	54	0,3	1939	82	0,37	2132	86	0,36	21	6	0,27	81	14	0,24	286	30	0,46	391	38	0,99
***Sj***	144	33	< 0.01	440	54	< 0.01	1024	82	< 0.01	1063	86	< 0.001	18	6	0,55	57	14	0,89	155	30	0,07	229	38	0,02

A one-sample Wilcoxon signed-rank test was used to compare Group 1 and Group 2 values to invasive-species values considered as reference for the comparison. V statistic values, sample sizes (n) of the Group and p values are provided for each comparison performed; df: 1 for all comparisons. Ig V, violet morph of *Impatiens glandulifera;* Ig P, pink morph of *Impatiens glandulifera;* Ig W, violet morph of *Impatiens glandulifera; Bd, Buddleja davidii; Rj, Reynoutria japonica;* Sj, Spiraea japonica. Values in red indicate significant P values.

Taken together, these results indicate that the four invasive species considered in our study use different visual advertising strategies when compared to their surrounding native species (Group 1): in general terms, they tended to be more salient to the bee eye either in one, two or in the three visual variables considered, which may facilitate their visual detection from farther distances and their learning based on chromatic cues associated with nectar reward. Only the white morph of *I. glandulifera* seems to be in disadvantage with respect to native species, thus raising the question of the mechanisms used by this morph to compensate via other (non-visual) advertising mechanisms for this deficit. Similar trends were found for the comparisons between invasive species and native species of Group 2, although these were less consistent.

### 3.4 Question 2: are invasive flowers species discriminable from native species to the bee eye?

Invasive species had higher achromatic and chromatic contrasts as well as higher spectral purity, which facilitate visual detection and learning by bee pollinators; yet, to what extent bees perceive them as chromatically similar to surrounding native species (i.e. Group 1) needs to be addressed separately. We thus determined the color similarity between invasive and native species, which can be estimated based on the distance between color loci in the color hexagon. We used the threshold value of 0.1 hexagon units (HU) reported for colour discrimination of bees trained under absolute conditioning (Dyer and Chittka, 2004) and determined if the color distance between the invasive species considered in our work and Groups 1 and 2 of native species was above or below this threshold.

The global analysis (i.e. taking all invasive species together) showed no significative differences of variance homogeneity within the three groups (one-way ANOVA: F2,78 = 2.21, p = 0.11). The between-group comparison showed significant differences of color distance between Groups 1 and 2 (PERMANOVA: F2,78 = 86.6, p < 0.01) and between the Invasive group and Group 2 (PERMANOVA: F2,78 = 13.8, p < 0.01). Thus, both Group 1 and 2 and Group 2 and the Invasive group appeared clearly distinct to bees. No significant difference was found between the Invasive group and Group 1 (PERMANOVA: F2,78 = 0.04, p = 0.14), thus suggesting that taken together, invasive species tended to be chromatically similar to plant species in Group 1 and different from plant species in Group 2.

**Figure 6:**
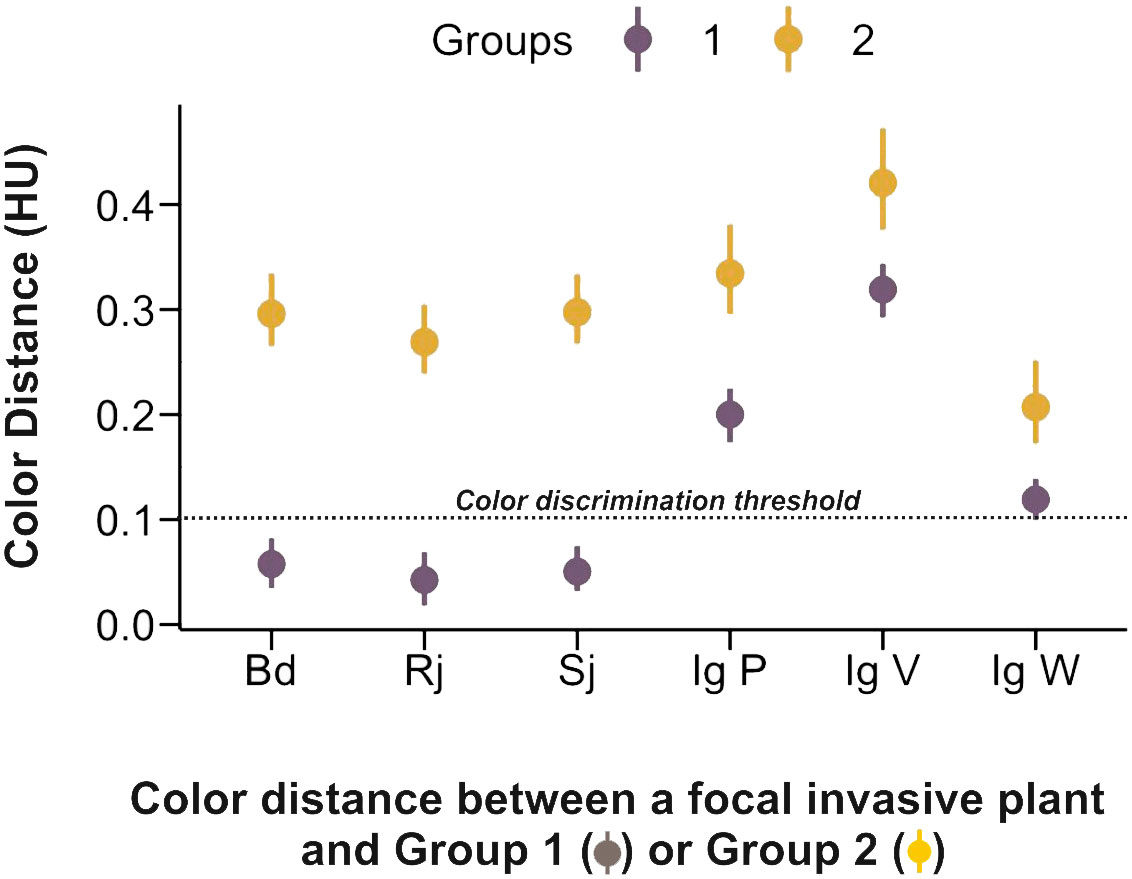
Color similarity (color distance in hexagon units, HU) between the invasive species and Group 1 and Group 2 of native species. The dashed line at 0.1 HU indicates the color discrimination threshold reported for bees trained under absolute conditioning (Dyer and Chittka, 2004), which corresponds to the ecological scenario of a color-constant foraging bee. All invasive species differed in color from the native species of Group 2. Three invasive species, *Buddleia davidii*, *Reynoutria japonica* and *Spiraea japonica*, were undistinguishable from the surrounding species of Group 1 in terms of color. The white morph of *Impatiens glandulifera* was separated from native species of Group 1 by a distance that was at the threshold of discrimination. The two other morphs of *I. glandulifera* (pink and violet) were clearly distinguishable from native species of Group 1.

As invasive species differ in their color display strategies, we refined the analysis of color similarity by comparing the color distance (HU units) between each invasive species/morph and Groups 1 and 2 of native species. **Figure 6** confirms that honey bees should be able to discriminate all four invasive species from the native species of Group 2 as the color distances separating them are well above threshold. However, the situation changes when the comparison is between invasive species and Group 1 of native species, which are precisely those surrounding principally the invasive species considered. Indeed, *B. davidii*, *R. japonica* and *S. japonica* are likely to be chromatically undistinguishable from the average color of Group 1 species as their mean color distance, lower and upper limits of the 95% confidence interval were 0.06, 0.03, 0.08 (*B. davidii*), 0.04, 0.02, 0.06 (*R. japonica*) and 0.05, 0.03, 0.07 (*S. japonica*). The white morph of *I. glandulifera* exhibited a similar trend although its values were at the threshold of discrimination (0,12, 0.10, 0.14) so that it is unclear whether it can be distinguished from Group 1 species in color terms. On the contrary, the highly salient violet and pink morphs of *I. glandulifera* are clearly distinguishable from Group 1 species in color terms (Ig V: 0.31, 0.29, 0.34; Ig P: 0.20, 0.18, 0.23), thus showing that the color-display strategies adopted by the four invasive species are not homogeneous. While the colors of three of them (*B. davidii*, *R. japonica* and *S. japonica*) favoured generalization with respect to surrounding native species, the highly salient *I. glandulifera* pink and violet morphs presented differentiable color displays.

## 4 Discussion

### 4.1 Visual signals of flowers as seen through the bee eye

Communication between plants and pollinators is essential for an effective pollination process (Proctor and Yeo, 1972; Barth, 1985). Throughout evolution, flowering plants have developed floral features aimed at pollinators to advertize feeding resources such as pollen and nectar. In exchange, flower-constant pollinators such as bees transport pollen grains to other flowers of the same species, thus enabling flower fertilization. In this partnership, visual signals play a fundamental role to guide pollinators to flowers from the distance (Kevan, 1978; Chittka and Menzel, 1992). Behavioral and physiological studies on honey bee vision, an insect with a model status for the analysis of perceptual phenomena (Giurfa and Menzel, 1997), have revealed that besides the fundamental role of color (i.e. dominant wavelength) displayed by flowers for advertisement, other parameter such as the achromatic contrast (the contrast of the visual target against the background evaluated through the L-receptor channel) and the spectral purity (the amount of a single wavelength component within a target reflection) are also used for visual orientation at different ranges. The achromatic contrast provided by a visual target enables farthest detection, i.e. when the targets subtend small visual angles between 5 and ca. 15° (Giurfa et al., 1996b; Giurfa et al., 1997) so that highest achromatic contrasts provide more visibility at the distance. At closer distances, i.e. at larger visual angles subtended by the targets, color (wavelength) information determines the choice of bees based on innate preferences in the first flights (Giurfa et al., 1995) and then on experience through the association of color and food reward (Menzel, 1985). In addition, spectral purity, which is the equivalent of color saturation, also guides the bees at even closer ranges, during the close-up recognition process. In this situation bees tend to follow the increasing gradient of spectral purity from the petal periphery to the center of the corolla and the enhanced spectral purity of nectar guides (Lunau, 1990; Lunau, 1992; Rohde et al., 2013), thus providing a direct orientation to the hidden location of reward within a flower.

Thus, given the importance of these visual signals for flower detection and recognition by pollinators, it is relevant to study to what extent the success of invasive plants relies on them in the competition with native species for pollinators using insect vision as an interpretational framework. In this study, we analyzed the color signals of four successful invasive species in the French Pyrenees and compared them with those of surrounding native species. Our analyses were performed from the perspective of honey bee vision in order to understand the strategies deployed by invasive plants to compete for pollinators and ensure a higher reproductive success. Clearly, honey bees are not the only pollinators of these plants, but they are among the major ones, and other frequent visitors such as bumble bees have a very similar set of color photoreceptors and a similar color vision (Peitsch et al., 1992).

Importantly, in this analysis we focused on chromatic and achromatic cues knowing that flower recognition may rely on further visual cues such as shape or symmetry (Lehrer et al., 1995; Giurfa et al., 1996a; Dafni and Kevan, 1997) and on non-visual cues such as scent (Dobson and Bergstrom, 2000; Friberg et al., 2014) or flower texture (Kevan and Lane, 1985), among others. Yet, in the case of bees, scent and spatial details contained in shape, as well as texture, operate in shorter, close-up ranges when compared with color cues, i.e. when the approach decision has been already made, as shown by studies by Karl von Frisch (1967). Thus, while these cues may divert a bee from inspecting an erroneous target once it has been chosen, the primary decision of approaching and choosing such a target is driven by cues such as the achromatic contrast and the chromatic contrast of a floral target, which were analyzed in our study.

### 4.2 The color similarity strategy: the case of *B. davidii, R. japonica* and *S. japonica*

The invasive species *B. davidii, R. japonica and S. japonica* shared a common chromatic identity with most native plant species, which exhibited blue and blue-purple flowers to the human eye (UV-blue, blue and blue-green to the bee eye; Group 1). These three invasive species clustered with Group 1 of native species (**Figure 3**), thus highlighting commonalities in terms of color properties. They were clearly different from other native species, which appeared yellow to the human eye (blue-green and green to the bee eye; Group 2). Interestingly, perceptual similarity correlated with adjacency. Group 1 species, which were similar to *B. davidii, R. japonica and S. japonica*, were more common in central sampling zones that constituted the immediate surrounding of an invasive species spot. On the contrary, Group 2 species, which differed from the three invasive species, were more frequent at the outermost zones of the sampling areas. Thus, the three invasive species shared similar colors with the native species that constituted their immediate surroundings and differed chromatically from distant native species. This suggests that invasive plants may profit from the established communication between pollinators and native plants to succeed in the invaded area, as reported for the invasive *Acacia saligna* in South Africa (Gibson et al., 2012). By adopting similar colors as their surrounding native plants, *B. davidii, R. japonica and S. japonica* may benefit from color generalization and thus attract pollinators from the distance. This hypothesis was further confirmed by the analysis of chromatic and achromatic contrasts, and spectral purity. These variables tended to be similar to those of native species (**Table 1**), thus indicating that the three invasive species were not perceptually salient among their surrounding native competitors. The analysis of color similarity, which provides a direct assessment of the bees’ capacity to discriminate between different color stimuli, showed that *B. davidii, R. japonica and S. japonica* were below the discrimination threshold when compared to the mean locus of Group 1 species (**Figure 6**). This results thus confirms that the color of these three invasive species could be confused or generalized with respect to that of the native species immediately surrounding them, a strategy that may increase their visitation rate and fertilization but also those of surrounding native species as the clustering of flowers with the same colors in the same area could attract more pollinators, a phenomenon termed ‘the magnet effect’ (Moeller, 2004; Peter and Johnson, 2008; Cuadra-Valdés et al., 2021).

Although the magnet effect can be advantageous for both the invasive and the native species depending on their fertility success, it could lead to more heterospecific (interspecific) pollen deposition, which could represent a waste of pollen grains (Morales and Traveset, 2008). This scenario could be detrimental for less frequent species as they could lose more pollen via the deposit on the wrong flowers. Thus, less abundant native species could suffer from the presence of invasive species in the area, given the abundance of the latter in the Pyrenees region.

### 4.3 The color salience strategy: the case of the pink and violet morphs of *I. glandulifera*

One of the invasive species, *Impatiens glandulifera*, presented floral color polymorphism, with white, pink and violet morphs, which appeared uv-blue/blue to the bee eye (**Figure 2**). The pink and the violet morphs did not cluster with the native species of Group 1 and even less with the native species of Group 2 (**Figure 3**). They thus differed significantly from all native species in their visual properties. For instance, they had significantly higher achromatic contrasts against the background when compared to surrounding native species of Group 1 (**Table 2**), which means that they were more detectable from the distance, at smaller visual angles, in terms of this achromatic variable, even before bees could perceive the color of the targets they were aiming at (Giurfa et al., 1996b; Giurfa et al., 1997). They also had significantly higher chromatic contrast and spectral purity than surrounding native species of Group 1 (**Table 1**), which means that their colors were more salient against the green background and their higher spectral purity rendered them more attractive for pollinators (Lunau, 1990; Rohde et al., 2013). Besides, their colors were dissimilar from those of native species of Group 1 and 2 (**Figure 6**), thus bestowing a unique, highly detectable and salient identity.

These characteristics may confer these *I. glandulifera* morphs with exceptional advantages in the context of bee pollination activities, which are governed by associative learning. In fact, flower constancy, the essential characteristic of bee foraging (Grant, 1950; Waser, 1986; Chittka et al., 1999) relies on the bees’ capacity to learn and memorize flower features such as the ones evaluated in our work based on their association with food reward (nectar or pollen) (Giurfa, 2007). Analyses of foraging activities using a Pavlovian learning framework have led to successful and valuable prediction of bee foraging activities (Greggers and Menzel, 1993; Montague et al., 1995). From these perspective, a basic tenet of Pavlovian associative learning refers to the salience of the stimuli to be learned and to the intensity of the reward delivered during learning trials (Rescorla and Wagner, 1972): salient stimuli increase the learning rate, leading to a faster reaching of a learning plateau. Similarly, better rewards facilitate learning. Thus, the pink and the violet morphs of *I. glandulifera* could eventually outcompete their surrounding native flowers via highly attractive and salient visual cues, which could be better learned than those of their native competitors, ensuring thereby efficient flower constancy. Additionally, *I. glandulifera* flowers have a particularly rich nectar, which is more rewarding than that of any known native plant in central Europe (Chittka and Schurkens, 2001), thus fulfilling the reward-intensity criterion required for improved associative learning.

In addition to nectar, pollen may also act as a reinforcing resource for bee pollinators (Muth et al., 2016). Quantitative information for pollen abundance and quality is not available to the best of our knowledge for the invasive species considered in our work. Yet, invasive pollen transport by Hymenoptera (honey bees and bumblebees) in field plots in which *I. glandulifera* coexisted with native species was significantly high as bees were found to carry more pollen from *I. glandulifera* than from native species (Lopezaraiza-Mikel et al., 2007). Thus, this invasive species may reinforce pollinator visits not only via particularly rich nectar but also via pollen.

Based on these features, the pink and violet morphs of *I. glandulifera* are, in principle, well equipped to tempt bee pollinators away from native flowers, potentially reducing thereby the fitness of native flora. Indeed, studies on the effects of both proximity and abundance of *I. glandulifera* on the reproductive success of native plant species showed that abundance of the invasive species led both honey bees and bumble bees to visit more often the invasive species to the detriment of the native ones (Cawoy et al., 2012). These arguments should, nevertheless be considered with caution in the absence of fitness studies focusing on the native and the invasive plants in the study community and because visual signals are not the only ones predicting the reproductive success of insect-pollinated plants.

### 4.4 The case of the white morph of *I. glandulifera*

The white morph of *I. glandulifera* represents an interesting case in visual terms as it is neither well detectable against the background (**Table 2**) nor well distinguishable from its surrounding native plants as the color distance separating them is at the threshold of discrimination (**Figure 6**). The flowers of this morph presented significantly lower achromatic contrast and chromatic contrast against the background and lower spectral purity (**Figure 5**). Its flowers appear white to human eyes but not to bees as they do not reflect evenly along all the visible spectrum of bees, as shown in the inset of **Figure 2A** (Kevan et al., 1996); given the lack of reflection in the UV range, the flowers of this morph appear blue-green to bees. Less is known about these white morphs in terms of their natural pollinators. Also, reports on their relative abundance in different regions are scarce; however, when color morphs of *I. glandulifera* were quantified, white morphs co-occurred with the other morphs, yet being clearly less abundant (Valentine, 1971). Given their visual characteristics, it is tempting to suggest that white flowers of *I. glandulifera* rely on other, non-visual cues (e.g. scent), to attract pollinators. The lower chromatic distance to surrounding flowers (**Figure 6**) may promote approaches to the white flowers by pollinators foraging on surrounding native species, which would give them the opportunity to sense these cues and associate them with the rich nectar present in the white flowers. As our analyses revolved around honey bee vision, it could be possible that the main recipients of the white-morph signals are not bees but other animals endowed with a different visual system. The nature of non-visual advertisement in the white morph of *I. glandulifera* remains to be determined and constitutes a fascinating topic for future research.

## 5 Conclusions

Plant invasion relies not only on the competition for space, nutrients and sunlight with native species but also on the competition for local pollinators to enhance reproductive success and spread. Thus, a fundamental component for understanding the success of invasive plants is to evaluate their characteristics from the perspective of the sensory and cognitive abilities of pollinators, which are the main addressees of their signals. We adopted this perspective to analyze the case of four successful invasive plants in the French Pyrenees and evaluated their color signals using the extensive knowledge gathered on honey bee vision. By recording the spectral signals of these species as well as those of native species in the same areas in which the invasive plants are located, we could estimate the achromatic and chromatic salience of the invasive species and their color similarity to surrounding native species. In this way, we were able to distinguish the color advertising strategies employed by the invasive species and evaluate their contribution to their invasive success.

We showed that the four invasive species differ in their color advertising strategy. Three species (*R. japonica, S. japonica* and *B. davidii*) were generally inconspicuous against the background in achromatic and chromatic terms and could be hardly discriminated in terms of color from their immedialy surrounding native plants. These characteristics may promote generalization and potentially attract visits from a flower constant pollinator foraging on a similar native species. The remaining species, *I. glandulifera*, presented three morphs with different characteristics. The pink and the violet morph were highly salient in chromatic and achromatic terms against the background and were very different from their surrounding native species. These features provide a distinctive identity, which may facilitate their detection and learning in association with the rich nectar they provide, thus potentially endowing the plants with significant advantages in the competition for pollinators. The white morph, on the contrary, did not present salient visual features, thus raising the double question of the sensory channels it may use to advertise its presence and of the natural addressees of its signals.

Our study focused on honey bees as a main pollinator of the four invasive floral species considered in our work in the Pyrenees landscape. Yet, in the same environment, many other insect species visit the invasive species analyzed, thus raising the question of the generality of our findings with respect to the perceptual capacities of these alternative pollinators. Answering this question is difficult as for some insect species that visit these invasive flower species, photoreceptor types may not have been characterized by means of electrophysiological recordings, thus precluding perceptual modeling analyses and building of color spaces for which this information is mandatory. In the case of other hymenopterans such as bumble bees, which are also regular visitors of the four invasive species considered, the spectral sensitivity curves of their three photoreceptor types show high similarity with that of honey bees (Peitsch et al., 1992). Modeling analyses on the optimality of spectral sensitivity curves in Hymenoptera showed that the optimal color receptors in terms of their capacity to finely code flower colors invariably display peak sensitivities at wavelengths of 330, 430 and 530 nm (Chittka and Menzel, 1992). These values correspond to those of λ_max_ found both in honey bees and bumble bees as well as in many other hymenopterans for which photoreceptor sensitivities have been characterized by means of electrophysiological methods. Thus, the conclusions reported in our work may not differ substantially in the case of bumble bees and other trichromatic hymenopterans with similar photoreceptor sensitivities.

While the unveiling of the visual strategies used by invasive plant species enlightens some crucial aspects of their success, it is, however, obvious that such success does not rely exclusively on visual cues but depends on multiple sensory dimensions, physiological adaptations and reproductive specificities, among others. It is thus important to stress that other cues [e.g., odors (Suchet et al., 2011), taste (Bestea et al., 2021)] may be as relevant as the visual cues analyzed in our study for the process of flower attraction and recognition and that their analysis should be based on the perceptual dimensions of the addressees. Overall, by adopting the perspective of the signal receiver, studies on biological invasion processes may uncover unknown aspects of the biology of invasive plants and contribute to the development of conservation strategies for native plants.

## Data availability statement

The datasets presented in this study can be found in online repositories. The names of the repository/repositories and accession number(s) can be found below: https://github.com/martindessart/Invasive_plants_through_bee-eye_Pyrenees.

## Author contributions

MD: Formal analysis, Methodology, Writing – review & editing. JA: Formal analysis, Methodology, Writing – review & editing. ET: Conceptualization, Methodology, Writing – review & editing. SG: Conceptualization, Funding acquisition, Supervision, Writing – review & editing. MG: Conceptualization, Funding acquisition, Supervision, Writing – original draft.

## Supplementary material

Supplementary Table 1 Relative abundance of invasive flowers. Patches labelled from A to L represent the 12 sampling sites. ‘Core’, ‘inner edge’, ‘outer edge’ and ‘surrounding’ refer to areas of each patch for sampling (see **Figure 1J**, main text). Numbers indicate the percentage of relative abundance measured in the field. See main text for details.

Supplementary Table 2 Flower and leave reflectance spectra included in our analyses. Reflectance spectra were obtained from 50 native species (n = 333 spectra; 1 spectrum corresponding to 1 flower part) and from the 4 invasive species mentioned above (n = 102 spectra), all collected in the field (left column, “measured in the field”). The FRED database (http://www.reflectance.co.uk/) allowed us to include 16 additional native species (n = 34 spectra) to complement our measurements (middle column, “measured in the field”). These species were present in the field and were counted for abundancy measurements but were inaccessible for reflectance measurements. The same database was used to include complementary measurements for 32 native species (n = 74 spectra) that were also sampled in the field. Overall, our analyses included 543 spectra (435 from the field and 108 from the FRED data) from 70 plant species, including the 4 invasive species (bottom of left column), which were the focus of our study, and 66 native species (18 exclusively from the field, highlighted in green, 16 exclusively from the FRED data base, highlighted in yellow) and 32 both from the field and the FRED data base; not highlighted). The spectral reflectance from leaves of 15 species (n = 25 spectra) was also measured to characterize the green foliage background (right column, “leave reflectance spectra”).

Supplementary Table 3 Chromatic contrast, achromatic contrast and spectral purity dataset. ‘Group’ refers to the three categories used for analyses: ‘Invasive’, which indicates an invasive species, ‘Group 1’ and ‘Group 2’, which refer to the two groups of invasive plants defined through clustering analysis (see **Figure 3**, main text). The three morphs of *I. glandulifera* (P = pink, V = violet and W = white) are indicated in separate lines.
