# Supplementary material for: Color-advertising strategies of invasive plants through the bee eye
Source: Front Plant Sci. 2024 May 22;15:1393204. doi: 10.3389/fpls.2024.1393204 (PMC11150686; doi:10.3389/fpls.2024.1393204)
Supplement: Supplementary Table 1 — Relative abundance of invasive flowers. Patches labelled from A to L represent the 12 sampling sites. ‘Core’, ‘inner edge’, ‘outer edge’ and ‘surrounding’ refer to areas of each patch for sampling (see Figure 1J , main text). Numbers indicate the percentage of relative abundance measured in the field. See main text for details. [file DataSheet_1.zip › CAPTIONS FOR SUPPLEMENTARY TABLES.docx]

**CAPTIONS FOR SUPPLEMENTARY TABLES**

**Table S1: Relative abundance of invasive flowers.** Patches labelled from A to L represent the 12 sampling sites. ‘Core’, ‘inner edge’, ‘outer edge’ and ‘surrounding’ refer to areas of each patch for sampling (see Fig. 1J, main text). Numbers indicate the percentage of relative abundance measured in the field. See main text for details.

**Table S2: Flower and leave reflectance spectra included in our analyses.** Reflectance spectra were obtained from 50 native species (n = 333 spectra; 1 spectrum corresponding to 1 flower part) and from the 4 invasive species mentioned above (n = 102 spectra), all collected in the field (left column, “measured in the field”). The FRED database (http://www.reflectance.co.uk/) allowed us to include 16 additional native species (n = 34 spectra) to complement our measurements (middle column, “measured in the field”). These species were present in the field and were counted for abundancy measurements but were inaccessible for reflectance measurements. The same database was used to include complementary measurements for 32 native species (n = 74 spectra) that were also sampled in the field. Overall, our analyses included 543 spectra (435 from the field and 108 from the FRED data) from 70 plant species, including the 4 invasive species (bottom of left column), which were the focus of our study, and 66 native species (18 exclusively from the field, highlighted in green, 16 exclusively from the FRED data base, highlighted in yellow) and 32 both from the field and the FRED data base; not highlighted). The spectral reflectance from leaves of 15 species (n = 25 spectra) was also measured to characterize the green foliage background (right column, “leave reflectance spectra”).

**Table S3: Chromatic contrast, achromatic contrast and spectral purity dataset.** ‘Group’ refers to the three categories used for analyses: ‘Invasive’, which indicates an invasive species, ‘Group 1’ and ‘Group 2’, which refer to the two groups of invasive plants defined through clustering analysis (see Fig. 3, main text). The three morphs of *I. glandulifera* (P = pink, V = violet and W = white) are indicated in separate lines.
